# Differential interaction of the dark septate endophyte *Cadophora* sp. and fungal pathogens *in vitro* and *in planta*

**DOI:** 10.1093/femsec/fiz164

**Published:** 2019-10-14

**Authors:** Wael Yakti, Gábor M Kovács, Philipp Franken

**Affiliations:** 1 Leibniz Institute of Vegetable and Ornamental Crops, Theodor-Echtermeyer-Weg 1,14979 Großbeeren, Brandenburg, Germany and Institute of Biology, Plant Physiology Department, Humboldt University of Berlin, Philippstraße 13, 10115 Berlin, Germany; 2 Department of Plant Anatomy, Institute of Biology, Eötvös Loránd University, Pázmány Péter sétány 1/C, 1117 Budapest, Hungary; 3 Plant Protection Institute, Centre for Agricultural Research, Budapest 1022, Hungary; 4 Erfurt Research Centre for Horticultural Crops, University of Applied Sciences, Kühnhäuser Straße 101, 99090 Erfurt, Thuringia, Germany

**Keywords:** dark septate endophytes, fungi, plant pathogens, *Cadophora*, biocontrol

## Abstract

Dark septate endophytes (DSEs) present a group of widespread root-colonizing fungi. The role of these endophytes in ecosystems and their interactions with plant pathogens are not well understood. In the current study, we assessed the antagonistic potential of the model DSE *Cadophora* sp. against the tomato soilborne pathogens *Rhizoctonia solani*, *Pythium aphanidermatum* and *Verticillium dahliae*. To investigate their interactions, we conducted *in vitro* assays followed by a greenhouse experiments in which tomato plants were inoculated with different combinations of the DSE and pathogens. RNA accumulation of selected tomato pathogenesis-related genes and of *Cadophora* sp. genes with putative antifungal function was analyzed. *Cadophora* sp. inhibited the growth of the fungal pathogens *in vitro* and vice versa; a negative impact of the pathogens on the growth of the DSE was also detected. In roots, however, this mutual negative interaction could not be observed. Expression analyses of plant genes could not explain this differential effect, but among the *Cadophora* sp. genes analyzed, a gene coding for a chalcone synthase was downregulated *in planta*. The data indicate that plants can change the interaction between fungi and, therefore, *in vitro* detected antagonism does not necessarily reflect the situation inside the plant.

## Teaser

*In vitro* detected antagonism does not necessarily reflect the situation *in planta*.

## INTRODUCTION

Fungal plant pathogens are detrimental factors in agriculture. They reduce crop production and consequently cause considerable economic losses (Fradin and Thomma 2006). Fungal pathogens are mainly controlled by the application of fungicides, about which concerns are growing for not only their environmental damage but also their effects on human health (Soares and Porto 2009), besides the increasing emergence of fungicide-resistant pathogens.

Microbes can protect plants from pathogens by enhancing plant resistance or tolerance, or by their direct antagonism toward plant pathogens. The enhancement of plant resistance or tolerance can be achieved when microbes alter plant biochemistry (Thomma *et al*. 2001; Caplan, Padmanabhan and Dinesh-Kumar 2008). Plant recognition of non-pathogenic microbes activates signaling pathways (cascades), which finally lead to enhanced plant resistance. Plant tolerance is often increased by the activation of antioxidative activities during colonization by non-pathogenic microbes (White and Torres 2010). This does not lead to lower pathogen infection but reduces symptoms development. Direct antagonism involves different mechanisms including competition for iron and other nutrients, parasitism by the secretion of proteins (e.g. enzymes) and the secretion of secondary metabolites with antibiotic activity (Heydari and Pessarakli 2010).

Numerous studies have spotted the use of microbes to control soilborne diseases but the number of available products does not meet the market's need (Alabouvette *et al*. 2009). In many cases, the effectiveness of microbes in protecting plants cannot be confirmed in practice (Ojiambo and Scherm 2006). Therefore, understanding the interactions between plant roots, beneficial microorganisms and soilborne pathogens, and how these interactions are influenced by environmental factors are of high importance for the future selection of effective biocontrol agents.

Endophytic fungi colonize plant tissues without causing symptoms, and exhibit a wide spectrum of interactions with their host plants (Saikkonen *et al*. 1998). Generally, fungal endophytes benefit plant performance in different ways. This includes the enhancement of plant growth through nutrient acquisition (Jumpponen 2001; Mandyam and Jumpponen 2005; Mahmoud and Narisawa 2013), and through the production of phytohormones (Schulz *et al*. 1998; Schulz *et al*. 2002; Schulz and Boyle 2005). Besides increasing tolerance and resistance of plants to abiotic and biotic stress, fungal endophytes can produce antimicrobial secondary metabolites inhibiting the growth of pathogens (Yu *et al*. 2010).

Dark septate endophytes (DSEs) are an abundant and widely distributed group of fungal root colonizers, belonging to the Class 4 of non-clavicipitaceous fungal endophytes (Rodriguez *et al*. 2009). They are known for their septated and melanized hyphae, and their ability to form microsclerotia in plant roots. The role of DSEs in ecosystem functioning is still ambiguous (Mandyam and Jumpponen 2005) and knowledge about their effects on plants is scarce. Inoculation experiments were carried out with several DSE species to improve the understanding of their interaction with their host plant; some have shown to positively affect tomato roots and fruit quality (Andrade-Linares *et al*. 2011), and to improve tomato growth under organic N conditions (Mahmoud and Narisawa 2013). Generally, an overall positive effect of DSEs on plants growing on sterile substrates was revealed by a meta-analysis (Newsham 2011).

Some studies have shown a potential for DSEs to control fungal plant diseases. The dark septate endophyte *Harpophora oryzae* protects rice plants from *Magnaporthe oryzae* by accumulating H_2_O_2_ and inducing plant systematic resistance (Su *et al*. 2013). *Phialocephala fortinii* reduced the symptoms caused by *Verticillium longisporum* in cabbage grown *in vitro* (Narisawa, Usuki and Hashiba 2004), *Leptodontidium orchidicola* decreased the wilting disease in tomato plants infected with *Verticilliumdahliae* (Andrade-Linares *et al*. 2011), and a *Cadophora* sp. (different than the one used in the present study) protected melon seedlings against *Fusarium oxysporum* f. sp. *melonis* in media supplied with an organic nitrogen source (Khastini *et al*. 2014). Furthermore, DSE fungi in the PAC group were shown to reduce mortality and to prevent the infection of *Picea abies* seedlings challenged with different pathogens *in vitro* (Tellenbach and Sieber 2012; Terhonen, Sipari and Asiegbu 2016).

Tomato is one of the most consumed vegetables worldwide, whereas its global production has reached almost 165 million tons in 2013 (http://faostat.fao.org). With the availability of genomic data and many mutant lines (Sato *et al*. 2012), tomato is now considered a model plant (Kimura and Sinha 2008). Root diseases can have a huge impact on the production of tomato. *Rhizoctonia solani, Pythium aphanidermatum* and *Verticillium dahliae* are good examples of soilborne pathogens, which cause major yield losses to tomatoes and are difficult to control (Barbara 2003; Klosterman *et al*. 2009; Warner 2012).

In this study, we investigated the 3-fold interaction between the DSE *Cadophora* sp., tomato plants and the three distinct pathogens: the oomycete *P. aphanidermatum* (phylum: Heterokontophyta), the true fungi *R. solani* (phylum: Basidiomycota) and *V. dahliae* (phylum: Ascomycota). The DSE *Cadophora* sp. was chosen because its genome sequence is available (Knapp *et al*. 2018). Together with the genome data of at least two of the three pathogens (Klosterman *et al*. 2011; Wibberg *et al*. 2015) and of the model tomato (Sato *et al*. 2012), it will be possible to study the molecular mechanisms underlying the interactions in the future. *In vitro* assays as well as greenhouse inoculation experiments were conducted to assess the biocontrol potential of the DSEs model *Cadophora* sp. Growth of the interacting fungi was monitored by morphological parameters (*in vitro*) or by quantitative PCR (*in planta*). We hypothesize that the presence of *Cadophora* sp. in natural ecosystem is accompanied by its ability to compete with other fungi, some of which are phytopathogenic. A second hypothesis is that the restriction of pathogens’ growth can also be observed during the colonization of the host plant. The third hypothesis states that differential interaction of DSEs with pathogenic fungi inside and outside the plant is based on differences in plant and/or fungal gene expression.

## MATERIALS AND METHODS

### Fungal strains

*Cadophora* sp. DSE1049 (Knapp, Pintye and Kovacs 2012) was maintained on a complete medium (Pontecorvo *et al*. 1953). The phytopathogenic fungus *V. dahliae* TomIGZ (accession GU060637, Race 1) *R. solani* AG3PT isolate Ben3 and *P. aphanidermatum* (BBA 70417) were obtained from the strain collection of Leibniz Institute for Vegetables and Ornamental Crops, Großbeeren, Germany. The pathogens were maintained on PDA medium and used in the experiments.

### *In vitro* antagonism test between DSEs and fungal pathogens

*Cadophora* sp. was tested for its antifungal activity against the pathogens *R. solani*, *P. aphanidermatum* and *V. dahliae* on a solid medium. Twenty-five percent PDA plates were inoculated with 5 mm diameter plugs of *Cadophora* sp. at one side, and a pathogen plug on the other side allowing a distance of ∼6 cm between the two plugs. The pathogens and *Cadophora* sp. were grown in axenic cultures as a control, and each combination was repeated four times. Plates were inoculated with *Cadophora* sp. and *V. dahliae* at the same time, whereas in the case of *R. solani* and *P. aphanidermatum*, due to their faster growth, *Cadophora* sp. was transferred to the media first, incubated at 26°C for 5 days to allow the colonies to get established and thereafter the pathogens plugs were introduced. The colonies were cultivated until the pathogens have covered all the control plates (in the case of *R. solani* and *P. aphanidermatum*) or when no further growth alterations were observed in the dual culture (in the case of *V. dahliae*). The radii of fungal colonies were measured toward the center of the opposing colony and compared to the radii of the same species in the axenic culture. The viability of pathogens in the *Cadophora* sp. confronted mycelium was tested by transferring a 2 mm fungal plug from the ‘contact zone’ and plating it on new agar plates.

### The effect of secreted metabolites on the growth of the phytopathogenic fungi

*Cadophora* sp. was inoculated into flasks containing 250 mL complete medium, and was shaken at 120 rpm for 3 weeks at 27°C. Fungal mycelia were removed by simple filtration and 100 mL of the filtrate was extracted with 2× the amount of ethyl acetate and concentrated to a volume of 5 mL using a rotary evaporator (KNF, Trenton, New Jersey). Five millimeter filter paper disks were immersed in the extract or in pure ethyl acetate as a control. Ethyl acetate was left to evaporate from the filter papers under the clean bench, and one extract-immersed filter paper was put on the side of a 25% PDA Petri dish, with a control filter paper on the opposing side allowing 6 cm in between. Five millimeter plug of each pathogen was transferred to the center of the plate allowing a distance of 3 cm from each of the filter papers. Colonies were let to grow for 2 days in the case of *P. aphanidermatum*, 3 days in the case *R. solani* and 14 days in the case of *V. dahliae*. The growth of the fungal colonies toward the extract-immersed filter disk was measured and compared with the growth toward the control filter disk. The test was carried out in three replicates.

### Greenhouse pot experiments

The experiments were carried out in Großbeeren (52°N, 13°E) in greenhouse conditions (see below). The first experiment had 10–12 repetition per treatment and two factors ‘Pathogen’ (*R. solani*, *P. aphanidermatum*, *V. dahliae* or control) and ‘DSE’ (*Cadophora* sp. or control). Tomato seeds (*Solanum lycopersicum* cv. Hildares F1, Hild Samen GmbH, Marbach, Germany) were surface sterilized by soaking for 4 min in 2.5% NaClO, followed by washing with sterilized water for five times. Seeds were germinated on MS medium for 2 weeks in a growth chamber (Adaptis A1000, Conviron Germany GmbH, Berlin, Germany) set on 16 h day/8 h night at 23/21 °C, respectively, and 110 μmol m^−2^ s^−1^ light intensity, and were transplanted into pots containing 700 g per pot of quartz sand (RIGK GmbH, Wiesbaden, Germany) with 35% big particles (2 to 3 mm) and 65% small particles (0.5 to 1 mm). The seedlings were inoculated by drenching (see below) with DSEs or with a mock solution. The plants were challenged with the pathogens 2 weeks after DSE inoculation. In the first 2 weeks of the experiment, pots were irrigated daily with 50 mL of Hoagland's solution (De Kreij *et al*. 1997). Starting from the 3rd week, the daily supply of Hoagland's solution was increased by 50 mL every week reaching 300 mL on the 7th week. Pot saucers were kept full of osmotic water to ensure plant water supply. Plants were cultivated in the greenhouse for 6 weeks after inoculation with *Cadophora* sp. (4 weeks after inoculation with pathogens) before harvest. Fresh and dry biomass were measured, and the colonization rates of the pathogens as well as of *Cadophora* sp. were assessed using qPCR assays.

A second experiment was carried out where plants were inoculated with *Cadophora* sp. or a mock solution, and challenged with the pathogen *V. dahliae* or with a mock solution of autoclaved inoculum. Seeds were sterilized and germinated as previously described. The seedlings were transplanted on sand (particle size: 0.5 to 1 mm)/vermiculite (50/50) (RIGK GmbH, Wiesbaden, Germany) and inoculated with *Cadophora* sp. by drenching. Ten days after DSE inoculation, the plants were challenged with *V. dahliae* and were cultivated for 14 days (24 days in total). Fresh and dry shoot biomass were assessed. DNA was extracted from the roots to assess the colonization rate of *V. dahliae* as well as of the two DSEs using qRT-PCR. RNA was extracted from roots to assess the expression of tomato pathogenesis-related (PR) genes.

In the first greenhouse experiment, greenhouse day/night temperatures were 19.8/16.15°C with a relative humidity of 57/55% and a mean daily radiation of 33 mol m^−2^ day^−1^. In the second greenhouse experiment, temperatures were 25/20°C, relative humidity was 70/78% and a mean daily radiation was 45.7 mol m^−2^ day^−1^.

### Inoculation and inoculum preparation

*Cadophora* sp. inoculum was prepared by introducing three fungal plugs to 200 mL liquid complete medium (Pontecorvo *et al*. 1953) in a flask. Plugs were taken from the freshly grown mycelia on complete medium agar plates. Mycelia were grown for 3 to 4 weeks on a shaker (120 rpm) at 27°C and were collected through filtration, washed, suspended in distilled water and mixed for 45 seconds using a blender at minimal speed (Model D72, Moulinex, Leipzig, Germany). The number of propagules (spores and/or hyphal fragments) was calculated by spreading 1:100–1:10 000 dilutions of each suspension on complete medium agar plates supplied with ampicillin. The concentrations used were 0.9 × 10^5^ cfu/mL for the first greenhouse experiment and 3 × 10^5^ cfu/mL for the second. In the first experiment, plants were inoculated by drenching 10 mL of propagules suspension on the roots, while in the second experiment, 25 mL were drenched. In both experiments, an autoclaved suspension served as mock control.

*Verticillium dahliae* inoculation was prepared by introducing three fungal plugs into sucrose sodium nitrate (SSN) liquid medium (Sinha and Wood 1968) and was cultivated for 4 weeks. *Pythium aphanidermatum* inoculum was prepared by growing the fungus in carrot broth medium (50 g/L). The inoculum and inoculation density for both fungi were calculated as previously mentioned. *Verticillium dahliae* inoculation in the first greenhouse experiment was done by drenching 16 mL of 5 × 10^5^ cfu/mL around the roots, and in the second experiment by drenching 30 mL of 7.4 × 10^5^ cfu/mL. Plants were infected with *P. aphanidermatum* by drenching 20 mL of 6 × 10^4^ cfu/mL into each pot.

In case of *R. solani*, the inoculum was prepared by growing the *R. solani* strain on autoclaved barley kernels for at least 2 weeks, and inoculation was done by introducing six infected kernels to each pot at a depth of ∼5 cm.

### Quantification of fungal DNA in the roots

qRT-PCR-based assay was used to quantify fungal colonization of the roots. Fungal DNA of each of the studied species was quantified using species-specific primers (Table S1, Supporting Information) in a relative quantification method, in which the relative abundance was assessed with respect to tomato ubiquitin gene.

Total genomic DNA was extracted from the randomly sampled roots of three biological replicates per treatment in both greenhouse experiments, using DNeasy Plant Mini Kit (Qiagen, Hilden, Germany) following the manufacturer's instructions. The reactions were performed on a real-time PCR system (model 7500; ABI, Warrington, UK) in three technical replicates in 10 µL volume and composed of 5 µL 2× SensiMix SYBR® Low-ROX kit (Bioline, Luckenwalde, Germany), 600 nM each primer and 50−200 ng template DNA. The PCR programs comprised a 3, 5 or 10 min denaturation at 95°C followed by 40 cycles of 95°C/15 s, 64.5°C/30 s, 72°C/30 s for *Cadophora* sp., 95°C/15 s, 58.5°C/30 s, 72°C/45 s for *P. aphanidermatum*, 95°C/30 s, 57°C/30 s, 72°C/1 min for *R. solani*, 95°C/30 s, 66°C/30 s, 72°C/30 s for *V. dahliae* and 95°C/15 s, 60°C/1 min for the tomato ubiquitin gene. All programs were followed by a melting curve analysis (60–95°C) to insure the amplification of a single product.

Normalized fungal abundance was assessed using a ∆Ct method modified from De Coninck *et al*. (2012); ∆Ct = Ct tomato ubiquitin − Ct fungal primer. 2^∆Ct^ values were further used as indicators of fungal abundance and compared between treatments (Su *et al*. 2013).

### Expression of tomato pathogenesis-related genes

In order to test whether the colonization by *Cadophora* sp. and *V. dahliae* influence the RNA accumulation of selected plant defense genes, total RNA was extracted from root tissues of the second greenhouse experiment using the innuPREP Plant RNA Kit (Analytik Jena AG, Jena, Germany) following the manufacturer's protocol. DNA was removed by on-column digestion using the innuPREP DNase I Kit produced by the same manufacturer. The extracted RNA was quantified using NanoDrop1000 Spectrophotometer (Thermo Fischer, Dreieich, Germany), 1–2 µg were reverse transcribed into cDNA using M-MLV Reverse Transcriptase kit (Promega GmbH, Mannheim, Germany) in a 25 µL reaction following the supplier's instructions. One microliter aliquot of 50 times diluted cDNA served as template for every qRT-PCR reaction, which also contained 5 µL 2× SensiMix SYBR® Low-ROX kit (Bioline, Luckenwalde, Germany), and 600 nM of each primer. Six tomato pathogenesis-related genes were assayed (Table S2, Supporting Information) encoding for proteinase inhibitor II (*Pin2*), endochitinase (*P3*), pathogenesis-related protein 6 (*P6*), *ß*-1,3-glucanase (*Glu*), chitinase (*Chi*) and PR-5 protein (*NP24*). Actin and *GADPH* were used after showing high expression stability (see below). The reactions were performed in a Real–Time PCR System (Applied Biosystems, Warrington, UK) and performed in three technical replicates. The PCR programs comprised a 7 min denaturation at 95°C followed by 40 cycles of 95°C/15 s then 1 min at annealing temperature (*T*m) according to the primers as shown in Table S2 (Supporting Information). Reference genes expression stability was assessed by calculating the expression stability (*M*) value and coefficient of variation (CV) using Biogazelle qBase+ version 3.0 (Biogazelle, Zwijnaarde, Belgium—www.qbaseplus.com), with the chosen thresholds *M* < 0.5 and CV < 0.25. Using the same software, calibrated normalized relative quantity (CNRQ) values of the six target genes were obtained to be compared between treatments.

### Expression of *Cadophora* sp. genes *in vitro* vs. *in planta*

RNA accumulation of nine selected genes of *Cadophora* sp. was analyzed in an *in vitro* system and compared between fungal growth on a medium versus growth in plant roots.

The annotated *Cadophora* sp. genome (Knapp *et al*. 2018) was screened for genes of putative antifungal function. Genes were collected using the search terms ‘thaumatin’, ‘chitinase’ and ‘chalcone synthase’ in the MycoCosm resource (Grigoriev *et al*. 2014). The protein sequences of selected genes were screened for the presence of signal peptides using SignalP 4.1 server (Petersen *et al*. 2011) using standard eukaryotic settings. Three thaumatin-like proteins (TLPs), three chitinases (chi) and two chalcone synthase genes (CHS) were further analyzed (Table S3, Supporting Information).

*Cadophora* sp. was grown on solid MS medium supplied with 10% sucrose, or with tomato seedlings grown *in vitro* on carbon-free MS medium. Fifty milliliter MS media was poured in 580 mL round glass jars (WECK, Wehr, Germany) and autoclaved cellophane membranes were placed on the top of the media to allow the harvest of fungus and roots. Inoculation with *Cadophora* sp. was done by adding and spreading 100 µL of a propagule solution (4 × 10^5^ cfu/mL) on cellophane's surface. Four 12 days old tomato seedlings, germinated *in vitro* as described before, were transferred to the carbon-free MS medium to allow the growth of *Cadophora* sp. *in planta*. *Cadophora* sp. was grown in a growth chamber (12 h day/12 h night 20 °C and 317 µmol m^−2^ s^−1^ light intensity) for 12 days.

The propagules solution was prepared by adding distilled autoclaved water on a PDA plate fully covered with mycelium of *Cadophora* sp. A sterilized artist's brush according to Silman, Nelse and Bothast (1991) was used to scrub the mycelium and free the propagules/ spores. The solution was then filtered using two layers of cheese cloth to remove big hyphal fragments. The concentration was estimated by plating a series of dilutions as previously mentioned.

The growth of *Cadophora* sp. into the roots was confirmed microscopically after staining root fragments with wheat germ agglutinin-Alexa Fluor 488 (WGA-AF 488) (Molecular Probes, Karlsruhe, Germany) in root tissues stained with Congo red according to Deshmukh *et al*. (2006). RNA was extracted from medium grown and roots-colonizing *Cadophora* sp. using innuPREP Bacteria/Fungi RNA Kit (Analytik Jena AG, Jena, Germany) following the manufacturer's instruction in addition to DNA removal using innuPREP DNase I Kit. cDNA was synthesized as previously described. A dilution of 1/10 of roots cDNA and 1/100 of medium-grown *Cadophora* sp. served as templates for qRT-PCR reactions. The qRT-PCR programs comprised a 7 min denaturation at 95°C followed by 40 cycles of 95°C/15 s then 1 min at 60°C; primer sequences are shown in Table S3 (Supporting Information). Genes coding for fungal actin and β-tubulin were chosen as reference genes as they showed high stability (*M* < 0.5 and CV < 0.25). CNRQ values were obtained using Biogazelle qBase+ version 3.0 (Biogazelle, Zwijnaarde, Belgium—www.qbaseplus.com) as mentioned above.

### Primer design and validation

*Cadophora* sp. ITS-based primer pair was used for the assessment of its abundance; sequences of all fungal genes were obtained from the MycoCosm database (Grigoriev *et al*. 2014). Sequences for the tomato gene encoding an endochitinase (PR3; accession number: XM_004237785.3) and for the *Cadophora* sp. ITS (accession number: JN859258) were obtained from the NCBI database (Coordinators 2016). All primers were designed using the NCBI primer design tool (https://www.ncbi.nlm.nih.gov/tools/primer-blast/).

The specificity validation of the newly designed primers was done by running PCR reactions using pure DNA templates of the other fungi included in this study, as well as non-inoculated tomato roots obtained from the greenhouse as negative controls. The products of all newly designed primers were cloned into the pGEM®-T Vector (Promega, Mannheim, Germany) and sequenced (Eurofins Genomics, Ebersberg, Germany) to confirm the target sequences. Primers efficiency was determined for all qRT-PCR reactions using LinReg software, which uses the amplification data recorded during the PCR reaction (Ramakers *et al*. 2003).

### Statistical analyses

All statistical analyses were performed using STATISTICA program version 11 (StatSoft Inc., Tulsa, Oklahoma). The comparison between the growth of colonies in the *in vitro* experiments, as well as comparing the colonization rates were done using one way analysis of variance (ANOVA), followed by Tukey's test at *P* = 0.05. In the greenhouse experiments, two ways ANOVA (*P* = 0.05) was used for plant growth parameters, and for plant gene expression data to assess the impact of factors and their interaction. *T*-test at *P* = 0.05 was performed to compare the expression of *Cadophora* sp. genes *in planta* vs. *in vitro*. All data sets were subjected to Kolmogorov-Smirnov test for normality and Levene's test for the homogeneity of variance. Kruskal–Wallis test (*P* = 0.05) was used on parameters lacking homogeneity of variance based on Levene's test.

## RESULTS

### *In vitro* antagonism between *Cadophora* sp. and fungal pathogens, and the effect of crude extracts of the secreted metabolites on the growth of pathogenic fungi

**Figure 1.**
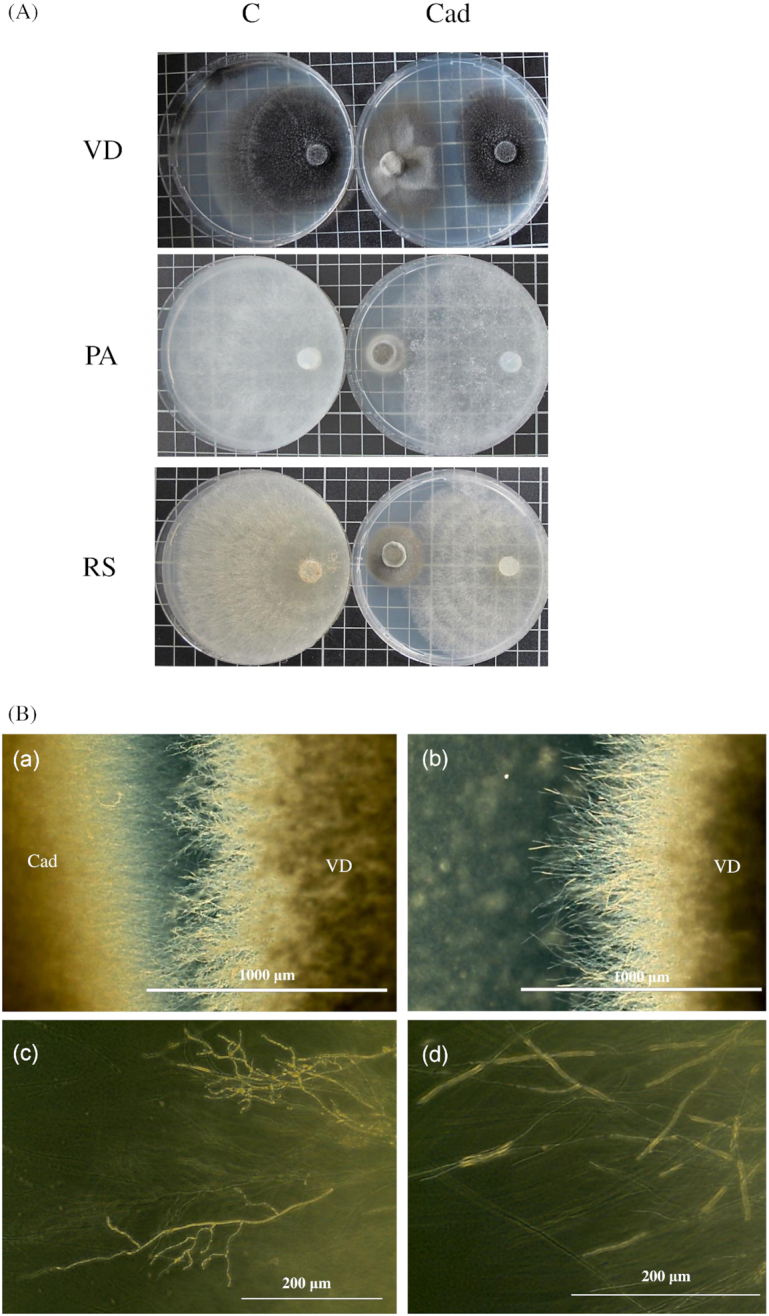
**(A)** The growth of fungal pathogens in the presence and absence of *Cadophora* sp. The three pathogens *V. dahliae* (VD), *P. aphanidermatum* (PA) and *R. solani* (RS) were grown in axenic cultures (C) or in dual cultures with *Cadophora* sp. (Cad). **(B)** The growth characteristics of *V. dahliae*’s hyphae. *Verticillium dahliae* growing on 25% PDA in the presence of *Cadophora* sp. (Cad) (a, c) or when growing alone (b, d).

**Figure 2.**
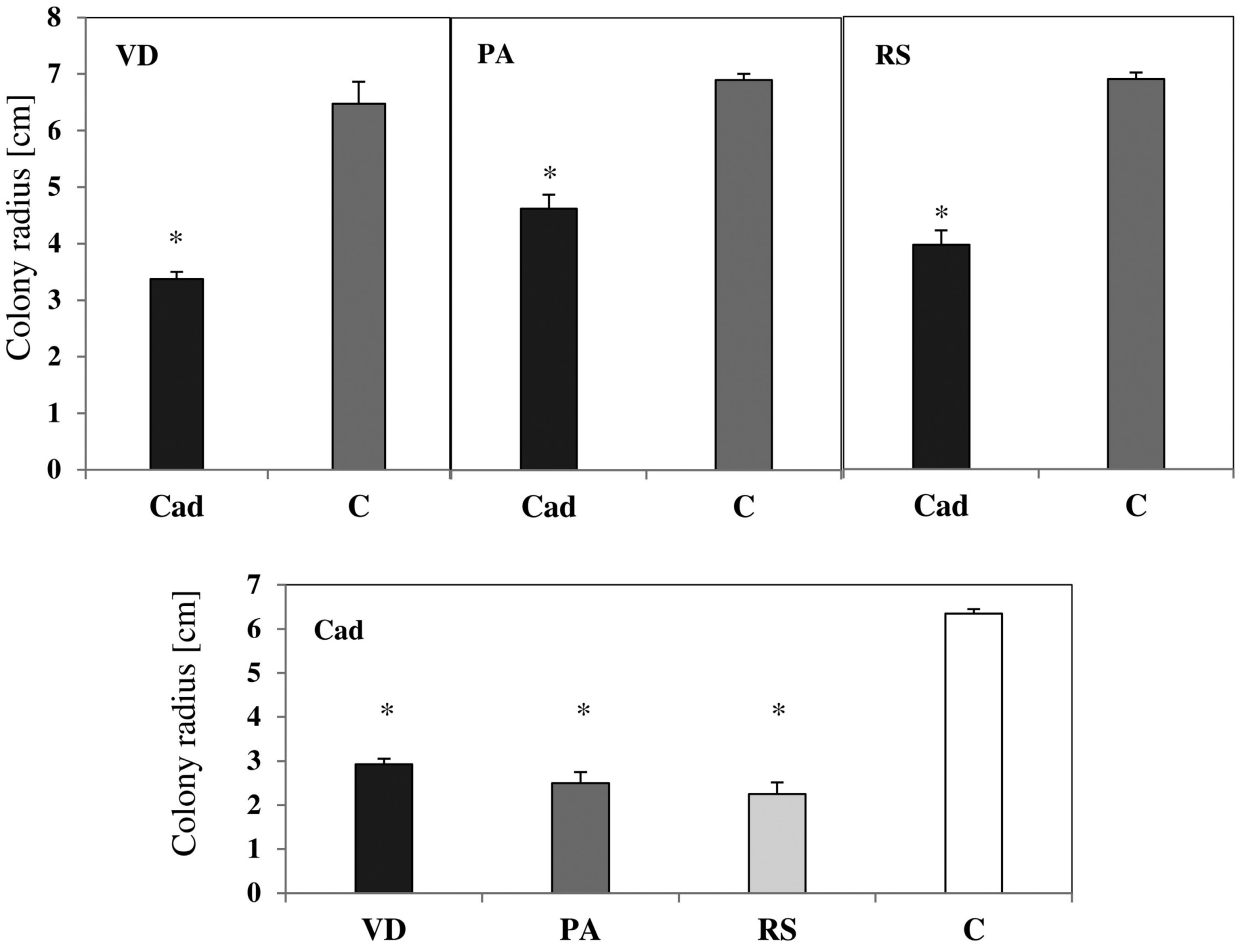
The growth inhibitory effects of *Cadophora* sp. and pathogenic fungi. The DSE *Cadophora* sp. (Cad) was grown in dual cultures with the three pathogens *V. dahliae* (VD), *P. aphanidermatum* (PA) and *R. solani* (RS). Each of the pathogens was grown in the presence and absence of *Cadophora* sp. (Cad). Shown are the means and standard deviations of the radii of fungal colonies measured toward the direction of the opposing colony in the co-culture, and compared to the growth of the same fungus in axenic culture (C). *T*-test (*P* = 0.05, *n* = 4) revealed an inhibitory effect of *Cadophora* sp. (Cad) on the growth of all pathogenic fungi. An inhibitory effect of pathogens on the radius of *Cadophora* sp. (Cad) was also observed. Significant differences between fungal growth in the axenic and the co-culture are indicated by asterisks.

In all combinations when the antagonism of *Cadophora* sp. with *R. solani*, *P. aphanidermatum* and *V. dahliae* was tested, an inhibition of fungal colonies was observed (Fig. 1A, Fig. 2). *Cadophora* sp. was able to diminish the growth of the three pathogens tested. On the other hand, the three pathogens significantly reduced the growth of *Cadophora* sp. colonies. When media plugs obtained from the confrontation line between the two colonies were transferred to a new PDA plate, all fungi emerged and were able to establish new colonies.

In addition to reduction in growth, *V. dahliae* exhibited alteration in hyphal morphology in the co-culture (Fig. 1B). Fungal hyphae tended to be more branched, curled and swollen in comparison to the control colonies. These hyphal growth patterns were observed in all species upon encountering an opposing colony in the co-culture but were less notable in comparison to *V. dahliae*.

The crude extract of *Cadophora* sp. altered the growth of *P. aphanidermatum* and *V. dahliae* colonies (Figure S1, Supporting Information), but this effect was not statistically significant.

### Greenhouse experiments

Some significant influences of the pathogens were detected on fresh and dry weights of shoots and roots in the greenhouse experiments. Plants growth was generally inhibited by the pathogens *P. aphanidermatum and R. solani* as they both significantly reduced shoot fresh and dry weight (Figure S2, Supporting Information), while *V. dahliae* only reduced shoot fresh weight (fresh weight is not shown) but not dry weight (Figure S2, Supporting Information). Concerning the roots, no effect on fresh and dry weights was observed, but a significant interaction was found between the factors ‘Cad’ and ‘pathogen’ for both parameters.

In the second greenhouse experiment, *Cadophora* sp. and *V. dahliae* exhibited no effect on plant growth (data not shown).

### Quantification of fungal DNA in the roots

**Figure 3.**
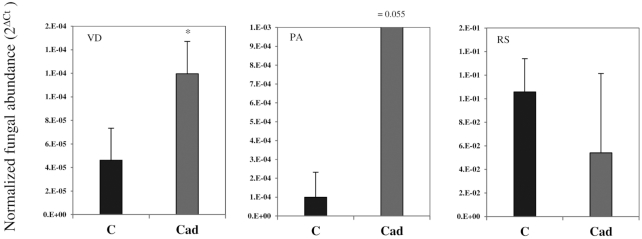
The colonization of tomato by pathogenic fungi. DNA was extracted from non-inoculated roots (C), or roots inoculated with *Cadophora* sp. (Cad), which were challenged with *V. dahliae* (VD), *P. aphanidermatum* (PA) or *R. solani* (RS). Pathogens were quantified using qRT-PCR assays and compared between treatments. *T*-test (*P* = 0.05, *n* = 3) was carried out and showed higher *V. dahliae* colonization in *Cadophora* sp.-inoculated roots. Significant differences in pathogens colonization rate between *Cadophora* sp.-inoculated and control plants are indicated by asterisks.

The pathogens were detected in all sampled inoculated plants. The colonization rates of *P. aphanidermatum* and *R. solani* did not significantly differ between *Cadophora* sp.-inoculated plants and controls. Colonization of tomato roots by *V. dahliae* was, however, significantly higher in *Cadophora* sp.-inoculated roots (Fig. 3). *Cadophora* sp. was quantified also to compare its colonization between the pathogen-infected roots and the controls and was found to be influenced by the pathogens (Figure S3, Supporting Information).

In the second greenhouse experiment, no significant effect on *V. dahliae* colonization in *Cadophora* sp.-inoculated roots was detected. In addition, the quantification of *Cadophora* sp. did not reveal any significant differences in colonization rates between *V. dahliae-*inoculated roots and the control treatment.

### RNA accumulation of tomato PR genes

**Figure 4.**
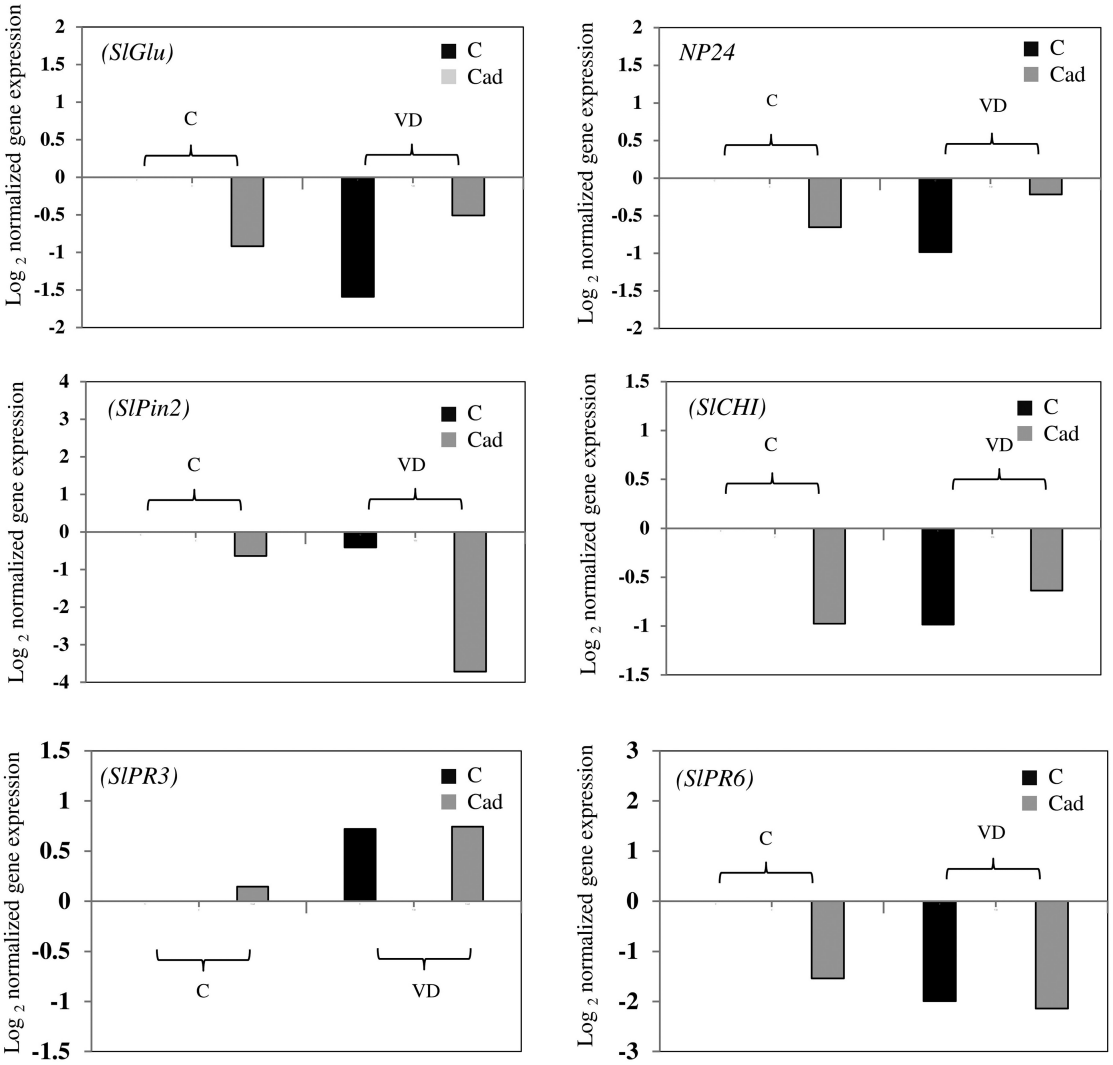
Normalized expression levels of defense-related genes in tomato. Tomato seedlings were not inoculated (C), or inoculated with *Cadophora* sp. (Cad) and challenged after 10 days with *V. dahliae* (VD) or were mock inoculated (C) (second greenhouse experiment) and grown for 2 weeks. RNA was extracted from roots and cDNA was synthesized. Shown are the Log_2_ CNRQ means of all treatments divided by that of control (C). Two ways ANOVA (*P* = 0.05, *n* = 3) on CNRQ values showed an impact of *V. dahliae* on the RNA accumulation of the endochitinase (*SlPR3*) gene. No other significant impacts were observed.

Our quantification showed that inoculation with *Cadophora* sp. did not influence the expression of the six genes analyzed, while colonization by *V. dahliae* resulted in the upregulation of the endochitinase gene *SlPR3* (Fig. 4). No interaction between factors was observed.

### Expression of *Cadophora* sp. genes *in vitro*vs.*in planta*

One hundred forty-three chitinase-encoding genes were found in the *Cadophora* sp. genome; 58 are putative secreted chitinases and contained a signal peptide. Three genes that contained carbohydrate-binding module 18 and a signal peptide were assessed. Three genes coding for thaumatin proteins were found in the *Cadophora* sp. genome and contained signal peptides. In addition, two chalcone/ stilbene synthase-encoding genes were found. These genes were also involved in the analysis.

RNA accumulation levels of genes encoding for thaumatin-like proteins (CdTLPs) and chitinases (CdChi) and one chalcone synthase gene (*CdCHS2*) were not differentially expressed between the two treatments. The chalcone synthase gene (*CdCHS1*) was significantly downregulated *in planta* in comparison to *in vitro*.

## DISCUSSION

### *Cadophora* sp.—pathogen interactions *in vitro*

*Cadophora* sp. was shown to suppress the growth of the tomato pathogens *P. aphanidermatum, V. dahliae* and *R. solani* belonging to distant evolutionary lineages (Fig. 1, Fig. 2). The *in vitro* suppression was accompanied with a clear inhibition zones between the colonies of *Cadophora* sp. and *V. dahliae*, which could be explained by the production of antibiotics or toxic metabolites (Swadling and Jeffries 1996). The suppression of fungal growth was not accompanied with killing the fungus during the experiment, as further growth was observed when plugs taken from the contact zones of the two colonies were transferred to a new plate. Fungi belonging to the genus *Cadophora* were scarcely investigated for the production of secondary metabolites (Almeida *et al*. 2010). A *Cadophora* fungus has been shown to produce isochromanones with antimicrobial activity (Rusman *et al*. 2015, 2018), and the isolate *Cadophora* sp. DSE1049 might be a good candidate for the production of antifungal compounds as in the dual cultures alterations in hyphal growth (e.g. intensive lateral branching) in all species were observed. Induced lateral branching and curling of hyphae are known fungal response to antibiotics (Baráthová, Betina and Nemec 1969; Gunji, Arima and Beppu 1983) and metabolites derived from dark septate endophytes were shown to induce such a morphogenesis (Terhonen, Sipari and Asiegbu 2016). Lateral branching has been found to be an avoidance strategy by the human pathogen *Aspergillus fumigatus* to prevent a contact with neutrophils (Ellett *et al*. 2017). In the case of *V. dahliae*, intensive branching and the observed alteration might be a strategy to reduce exposure to compounds produced by *Cadophora* sp. that are unfavorable for growth.

The crude extract of *Cadophora* sp. was shown to slightly alter fungal growth (Figure S1, Supporting Information), which indicated that bioactive secondary metabolites might have been produced in the liquid culture. An optimization of fungal growth conditions and crude extraction method may lead to the identification of antifungal secondary metabolites produced by *Cadophora* sp.

### DSE–pathogen interactions *in planta*, and impact on plant growth

The antagonism observed *in vitro* gave rise to the hypothesis that *Cadophora* sp. would be able to suppress the growth of pathogens *in planta*. Our first experiment showed a negative impact of the pathogens on plant growth and no impact of *Cadophora* sp. Positive effects of DSEs on plant growth have been mainly linked to improved plant nutrition (Haselwandter and Read 1982; Jumpponen, Mattson and Trappe 1998; Newsham 2000; Yakti *et al*. 2018; Yakti *et al*. 2019), for example, DSEs had significant effects on plant biomass when N was supplied in organic form (Newsham 2011) pointing out to their ability to solubilize nutrients. This could not be expected in our experimental system where all nutrients were supplied in their plant-available forms. Nevertheless, it is assumed that several factors, including environmental conditions and plant physiology, produce different host responses to endophytic colonization (Redman, Dunigan and Rodriguez 2001; Mandyam and Jumpponen 2015). Other studies have shown that DSEs can have negative impacts on different parameters of their host (Wilcox and Wang 1987; Tellenbach, Grünig and Sieber 2011; Reininger, Grünig and Sieber 2012; Terhonen, Sipari and Asiegbu 2016). In our experiments, *Cadophora* sp. had a negative effect on root biomass in the presence of *V. dahliae* which might be even an advantageous effect in the presence of a soilborne pathogen (Figure S2, Supporting Information).

In contrast to the *in vitro* observations, *Cadophora* sp. and pathogens did not show antagonistic effects on each other *in planta*. Moreover, plants inoculated with *Cadophora* sp. tended to have a higher level of *V. dahliae* colonization in the first greenhouse experiment (Fig. 3 ), combined with growth reduction in roots colonized by both fungi. In the case of *P. aphanidermatum*, a trend for more colonization was observed, but was not significant with a notably high standard deviation. Obtaining a representative root sample for the quantification of *P. aphanidermatum* is challenging (Kyuchukova *et al*. 2006), which might be an issue in our experiment, too.

To the best of our knowledge, the current study is the first to implement a qPCR assay to directly assess the interaction of DSE and pathogenic fungi in the roots. Tellenbach and Sieber (2012) have used quantitative PCR to assess only the abundance of the DSE *Phialocephala subalpina* with and without the presence of pathogenic oomycetes and found no effect of pathogens’ occurrence on the DSE's abundance, which is in accordance with our study. Whitaker and Bakker (2018) have also shown that *in vitro* antagonism of bacterial endophytes could not be observed in live plant tissues and pointed out that the commonly used *in vitro* assays cannot always predict*in planta* scenarios.

### The effect of co-inoculations on the expression of plant PR genes

The majority of studies that dealt with plant defense responses have been carried out with an infection of a sole pathogen or with co-inoculation with beneficial symbionts and fungal pathogens (Van Wees, Van der Ent and Pieterse 2008; Jiang, Zheng and Chen 2009; Jung *et al*. 2012; Muthamilarasan and Prasad 2013; Vos *et al*. 2013; Pusztahelyi, Holb and Pócsi 2015). Only limited knowledge is, however, available about the situation when more than one fungus is present showing negative effects in the plant (Tsror and Hazanovsky 2001; Abdullah *et al*. 2017). It has been observed that a plant-colonizing microbe could facilitate the invasion of a pathogen (Spoel, Johnson and Dong 2007). In one of these cases, it has been shown that a biotrophic fungus, *Albugo candida*, repressed the expression of defense-related genes allowing more microbial invasions (Cooper *et al*. 2008). We, therefore, hypothesized that the less negative or even positive interactions between the pathogens and the DSE could be due to a defense repression caused by the fungi. This hypothesis was tested for plants of the second greenhouse experiment, where control plants or plants inoculated with *Cadophora* sp. were challenged or non-challenged with *V. dahliae*. Genes for an endochitinase (*SlPR3*) and for the PR protein 6 (*SlPR6*) were selected as they have been upregulated in stems of *V. dahliae* infected plants (Robb *et al*. 2009). In the current study, only the expression of *SlPR3* was induced in roots infected with *V. dahliae*. Nevertheless, the hypotheses that DSEs could repress the expression of the selected genes had to be rejected.

### *Cadophora*sp. gene expression *in planta*vs.*in vitro*

**Figure 5.**
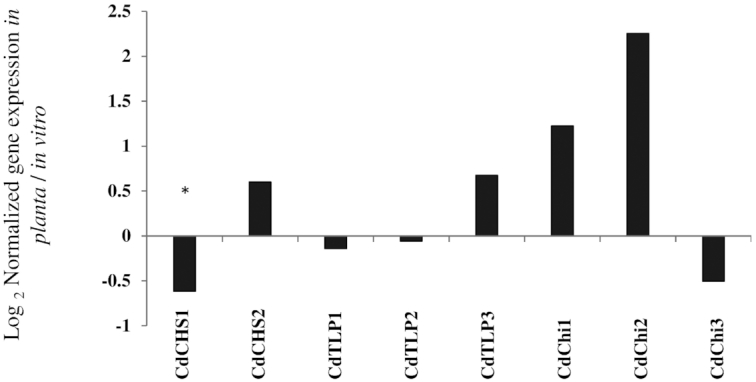
The expression of *Cadophora* sp. genes *in planta* vs. *in vitro*. *Cadophora* sp. was grown for 12 days on MS medium supplied with 10% sucrose (*in vitro* treatment), or in tomato seedlings grown on MS medium (*in planta* treatment). RNA was extracted and cDNA was synthesized. Three genes coding for thaumatin-like proteins (CdTLP1–3), three coding for lectins (CdChi1–3) and two genes coding for chalcone synthase (CdCHS1,2) were analyzed. Shown are the CNRQ values of *in planta* grown *Cadophora* sp. divided by the*in vitro* CNRQ values. *T*-test (*P* = 0.05, *n* = 3) was performed on CNRQ values and compared between treatments. Significant differences are indicated by asterisks.

Besides the effects of co-inoculation on plant defense, the lack of mutual suppression *in planta* compared to *in vitro* could be also based on differences in the direct interactions between the DSE and the pathogens. Even though the establishment of DSE colonization of the roots was allowed in the greenhouse experiments before plants were infected with the pathogen, no suppression of pathogenic growth was observed (Fig.   3). This gave rise to the hypothesis that the production of antifungal compounds by the DSE is downregulated *in planta*. To test this hypothesis, we grew *Cadophora* sp. *in vitro* and *in planta* and assessed the expression of genes putatively involved in the antagonistic activity of the fungus (Table S4, Supporting Information). Three secreted chitinase-encoding genes, three genes for thaumatin-like proteins (TLC) and two chalcone synthase-encoding genes (*CHS*) were selected to test the hypothesis by expression analyses. Chitinases hydrolyze chitin, a main component of fungal cell wall. In plants, chitinases play crucial role in defense against fungal pathogens (Punja and Zhang 1993), while in fungi they could have many functions including nutrition and morphogenesis (Adams 2004). TLCs are low molecular weight proteins that have been shown to be upregulated in plants in response to biotic stress (Singh *et al*. 2013). TLCs possess antibiotic activity against many fungal pathogens (Garcia‐Casado *et al*. 2000; Chu and Ng 2003; Ho, Wong and Ng 2007). CHS belong to the type III polyketide synthases which are involved in the production of a wide variety of bioactive secondary metabolites in microbes (Yu *et al*. 2012). In plants, it is a key enzyme in the biosynthesis of flavonoids (Knogge, Schmelzer and Weissenböck 1986; Dao, Linthorst and Verpoorte 2011) that has been shown to possess antimicrobial activity (Orhan *et al*. 2010; Mousa and Raizada 2013), playing a role in plant defense against pathogens. Nevertheless, no differences were found in the RNA accumulation levels of chitinases and TLPs, but one CHS was significantly downregulated *in planta* (Fig. 5). The results indicate a shift in the production of bioactive secondary metabolites by *Cadophora* sp. during its endophytic lifestyle. The products of the corresponding pathway in *Cadophora* sp. and their role in its antagonistic activity have to be tested.

## CONCLUSION

Compared to the other well-explored groups of root-associated fungi, the agricultural application potential of DSEs seems to be still enigmatic. DSEs possess high genetic and functional diversity, and their effects could vary based on the DSE–plant species combination and plant nutritional status (Mandyam and Jumpponen 2005; Newsham 2011; Mayerhofer, Kernaghan and Harper 2013). Therefore, more research is needed to build an overall idea about their function and potential application. It could be, however, shown in the current study that DSEs could have antagonistic activities, which is in clear contrast to arbuscular mycorrhizal fungi or other root-colonizing fungi where such activities could never be detected.

Our data reveal that the antagonism between the model DSE *Cadophora* sp. and pathogens observed *in vitro* could not be observed in greenhouse pot cultures. Nevertheless, different outcomes could be expected with different host plants, cultivation conditions or different pathogens. Expression data of plant defense-related genes did not provide an explanation because downregulation during co-inoculation was not observed. In contrast, the expression of a *Cadophora* sp. chalcone synthase encoding gene, a key enzyme in flavonoid biosynthesis in plants, showed a decreased expression *in planta*. Products of *Cadophora* sp.’s chalcone synthase, most probably fungal flavonoids as chalcone derivatives, might play a role in the observed antagonistic activity *in vitro* and the decreased mRNA accumulation of this gene *in planta* might have led to the loss of antifungal activity. Nevertheless, the antagonistic activity of *Cadophora* sp. may still be beneficial in terms of plant protection in a soil environment. While occupying soil niches and with their wide enzymatic arsenal (Mandyam, Loughin and Jumpponen 2010; Knapp and Kovács 2016; Knapp *et al*. 2018), DSEs might be able to limit the growth and persistence of plant-damaging fungi and prevent severe infestations. The ability of *Cadophora* sp. and other DSEs to produce antimicrobial metabolites in their saprophytic lifestyle in the soil is a topic for future research.

## Compliance with Ethics Requirements

This article does not contain any studies with human or animal subjects.

## Supplementary Material

fiz164_Supplement_Files
