# Supplementary material for: Differential interaction of the dark septate endophyte Cadophora sp. and fungal pathogens in vitro and in planta
Source: FEMS Microbiol Ecol. 2019 Oct 14;95(12):fiz164. doi: 10.1093/femsec/fiz164 (PMC6864363; doi:10.1093/femsec/fiz164)
Supplement: fiz164_Supplement_Files [file fiz164_supplement_files.zip › Yakti et al, Tables supplementary data.docx]

**Table 1.** Oligonucleotide sequences used for the quantification of fungi in the roots

| Primer name | Organism | Oligonucleotide sequence (5'-3') | | Target | [bp] | Reference |
| --- | --- | --- | --- | --- | --- | --- |
| Cspq1 Cspq2 | *Cadophora* sp. | for rev | CTAGAGCAAAGGATAGGCAGC CGAGAGGTTCGACGACTCTAA | ITS Region | 90 | This study |
| PaAsPyF PaAsAPH2B | *P. aphanidermatum* | for rev | CTGTTCTTTCCTTGAGGTG GCGCGTTGTTCACAATAAATTGC | ITS Region | 163 | (Asano *et al.* 2010) |
| RsITS U114  RsITS L396 | *R. solani* | for rev | GTTATTTTTTGTAATAAAATGATAATAAGTC ATCCTAATTGAGTTAACAAAAAGAT | ITS Region | 456 | (Genzel *et al.* 2018) |
| SlUbi3f SlUbi3r | Tomato | for rev | TCGTAAGGAGTGCCCTAATGCTGA CAATCGCCTCCAGCCTTGTTGTAA | Ubiquitin Gene | 119 | (Mascia *et al.* 2010) |
| VdS1  VdS2 | *V. dahliae* | for rev | CACATTCAGTTCAGGAGACGGA CCGAAATACTCCAGTAGAAGG | SCAR Region | 521 | (Li *et al.* 1999) |

**Table 2.** List of tomato primers used in the analysis of plant defense-related gene expression

| **Primer name** | **Gene encoding for** | **NCBI Accession** | **Oligonucleotide sequence (5'-3')** | | **Tm [°C]** | **Reference** |
| --- | --- | --- | --- | --- | --- | --- |
| SlPin2 | Proteinase inhibitor | AY129402 | for | TGATGCCAAGGCTTGTACTAGAGA | 61 | (Herman *et al.* 2007) |
|  |  |  | rev | AGCGGACTTCCTTCTGAACGT |  |  |
| SlPR3 | Endochitinase | XM_004237785.3 | for | AATTATGGTCCAGCAGGGCA | 61 | This study |
|  |  |  | rev | GGGCCAAATCCTTGTCCAGA |  |  |
| SlPR6 | Pathogenesis-related protein 1 | NM_001247385 | for | TCTTGTGAGGCCCAAAATTC | 61 | (Buhtz *et al.* 2017) |
|  |  |  | rev | CCAGCACCAGAATGAATCAA |  |  |
| SlGluA | 1,3-ß-glucanase | M80608 | for | TCAAACATCATGGCTACCTCACAA | 60 | (Fuentes-Silva *et al.* 2007) |
|  |  |  | rev | TCCCCATCATTCCATAACAAACAC |  |  |
| SlCHI | Chitinase | Z15140 | for | ATGGGGTTACTGTTTCCTTAGAGA | 60 | (Diaz-Perales *et al.* 1999) |
|  |  |  | rev | TGGCCCATAGTTGTAGTTGTGT |  |  |
| SlNP24 | Thaumatin-like protein | AF093743 | for | TGAAGGAATGGAATGGTGCT | 60 | (Kondo *et al.* 2001) |
|  |  |  | rev | TGAAAGAAGCCACAAAAGTTC |  |  |
| SlACT | Actin | BT012695 | for | GAAATAGCATAAGATGGCAGACG | 60 | (Løvdal and Lillo 2009) |
|  |  |  | rev | ATACCCACCATCACACCAGTAT |  |  |
| SlGAPDH | GAPDH | U93208 | for | ACCACAAATTGCCTTGCTCCCTTG | 60 | (Mascia *et al.* 2010) |
|  |  |  | rev | ATCAACGGTCTTCTGAGTGGCTGT |  |  |

**Table 3**. The list of *Cadophora* sp*.* primers used in the fungal gene expression analysis *in planta* vs. *in vitro.*

| **Gene** | **Annotation** | **Acc^.*^** | **Oligonucleotide sequence (5'-3')** | |
| --- | --- | --- | --- | --- |
| *CdTLP1* | Thaumatin-like protein | 159106 | for | CACATCCGCCAAAGCCGTCTG |
|  |  |  | rev | TCCGCCAAAATCGCCTTACTCATC |
| *CdTLP2* | Thaumatin-like protein | 613287 | for | CGTGCCCCTACATATGCGTCTCA |
|  |  |  | rev | ACCCCAAGTCATCTGCCACCTCA |
| *CdTLP3* | Thaumatin-like protein | 630200 | for | AAGGGCATTTCCAACGCATACG |
|  |  |  | rev | TTTTTCGGCTTCGCTCTTGTTACC |
| *CdChi1* | Chitinase | 567061 | for | AGCCGGCAAATCCCCTTCTACA |
|  |  |  | rev | TTCGGATCGCCCAAACACTCTC |
| *CdChi2* | Chitinase | 606975 | for | AATCCTCTCCCGTCGCAAGTTCA |
|  |  |  | rev | CTCCCAGACATTCGCCCACAG |
| *CdChi3* | Chitinase | 660056 | for | GGGGCATGCCATAGAGGAACG |
|  |  |  | rev | TGACAATCGGGAGCAGCACAATA |
| *CdCHS1* | Chalcone and stilbene synthases | 525783 | for | GCGGTGCGACGATCCTCTCAG |
|  |  |  | rev | GCGCAACCAACAACGAAATCCTT |
| *CdCHS2* | Chalcone and stilbene synthases | 543066 | for | CCCGAGAGTGCTGGAGTTTT |
|  |  |  | rev | TACCCCGAGTCCGATCAGTT |
| *CdAct* | Actin | 511595 | for | ACCATCCTCCATGAAGGTCA |
|  |  |  | rev | TCCGCTCTCGTCGTACTCTT |
| *CdTub* | Tubulin | 513701 | for | GGTTCGCTTGGTCTTCAGAG |
|  |  |  | rev | CCAACTCGGTCCAGGAGATA |

^*^Accession number from the MycoCosm database (<https://genome.jgi.doe.gov/programs/fungi/index.jsf> )

**References**:

Asano T, Senda M, Suga H et al. Development of multiplex pcr to detect five pythium species related to turfgrass diseases. *J Phytopathol* 2010;158: 609-15.

Buhtz A, Hohe A, Schwarz D et al. Effects of *Verticillium dahliae* on tomato root morphology considering plant growth response and defence. *Plant Pathol* 2017;66: 667-76.

Diaz-Perales A, Collada C, Blanco C et al. Cross-reactions in the latex-fruit syndrome: A relevant role of chitinases but not of complex asparagine-linked glycans. *J Allergy Clin Immunol* 1999;104: 681-7.

Fuentes-Silva D, Mendoza-Hernández G, Stojanoff V et al. Crystallization and identification of the glycosylated moieties of two isoforms of the main allergen Hev b 2 and preliminary X-ray analysis of two polymorphs of isoform II. *Acta Crystallogr F* 2007;63: 787-91.

Genzel F, Franken P, Witzel K et al. Systemic induction of salicylic acid-related plant defences in potato in response to *Rhizoctonia solani* AG3PT. *Plant Pathol* 2018;67: 337-48.

Herman M, Restrepo S, Smart C. Defense gene expression patterns of three SAR-induced tomato cultivars in the field. *Physiol Mol Plant Pathol* 2007;71: 192-200.

Kondo Y, Urisu A, Tokuda R. Identification and characterization of the allergens in the tomato fruit by immunoblotting. *Int Arch Allergy Immunol* 2001;126: 294-9.

Løvdal T, Lillo C. Reference gene selection for quantitative real-time PCR normalization in tomato subjected to nitrogen, cold, and light stress. *Anal Biochem* 2009;387: 238-42.

Li KN, Rouse DI, Eyestone EJ et al. The generation of specific DNA primers using random amplified polymorphic DNA and its application to *Verticillium dahliae*. *Mycol Res* 1999;103: 1361-8.

Mascia T, Santovito E, Gallitelli D et al. Evaluation of reference genes for quantitative reverse-transcription polymerase chain reaction normalization in infected tomato plants. *Mol Plant Pathol* 2010;11: 805-16.
