# Supplementary figures and images for: Differential interaction of the dark septate endophyte Cadophora sp. and fungal pathogens in vitro and in planta
Source: FEMS Microbiol Ecol. 2019 Oct 14;95(12):fiz164. doi: 10.1093/femsec/fiz164 (PMC6864363; doi:10.1093/femsec/fiz164)

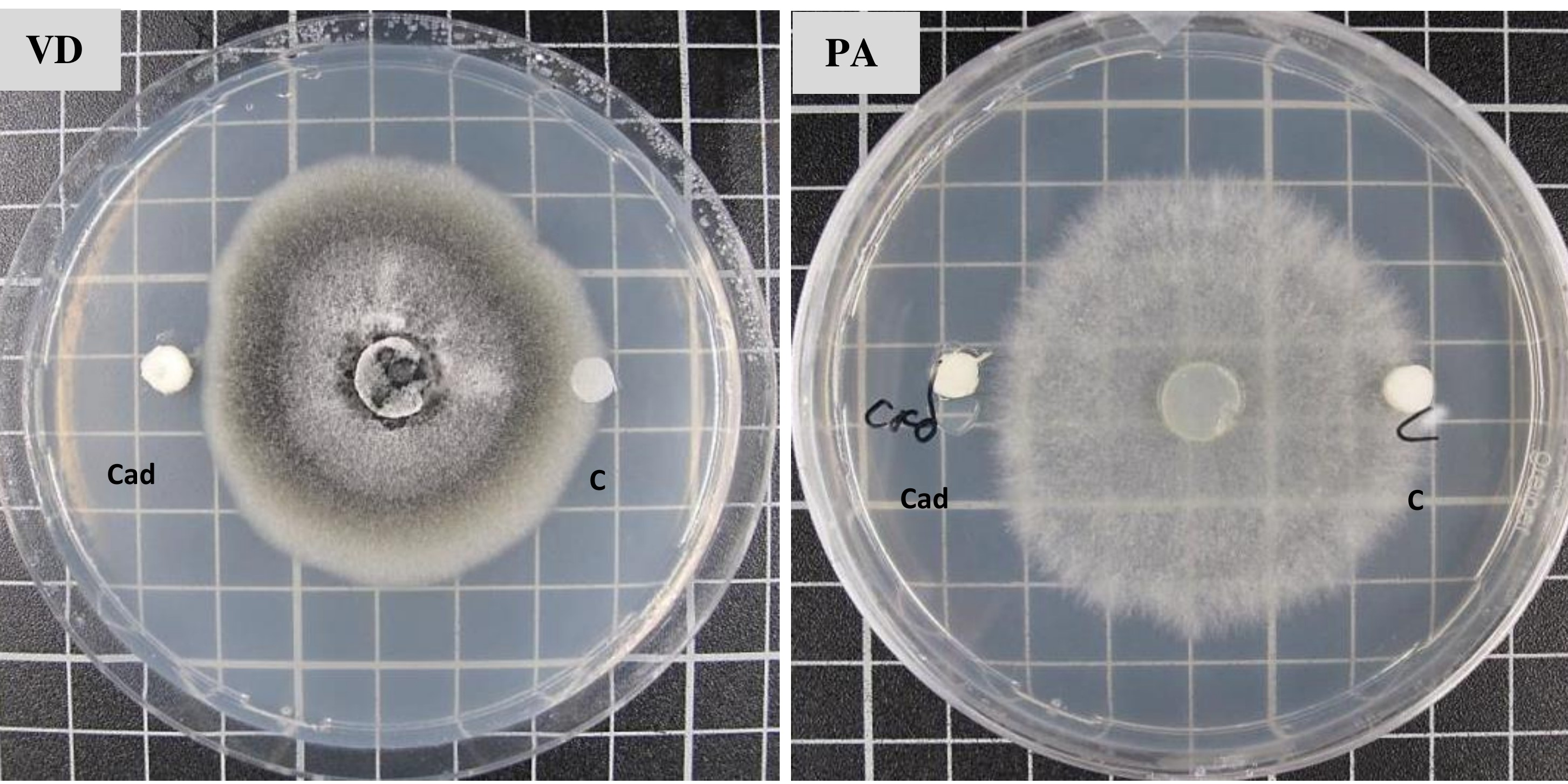

Supplement: fiz164_Supplement_Files [file fiz164_supplement_files.zip › Supplementary 2s.tif]

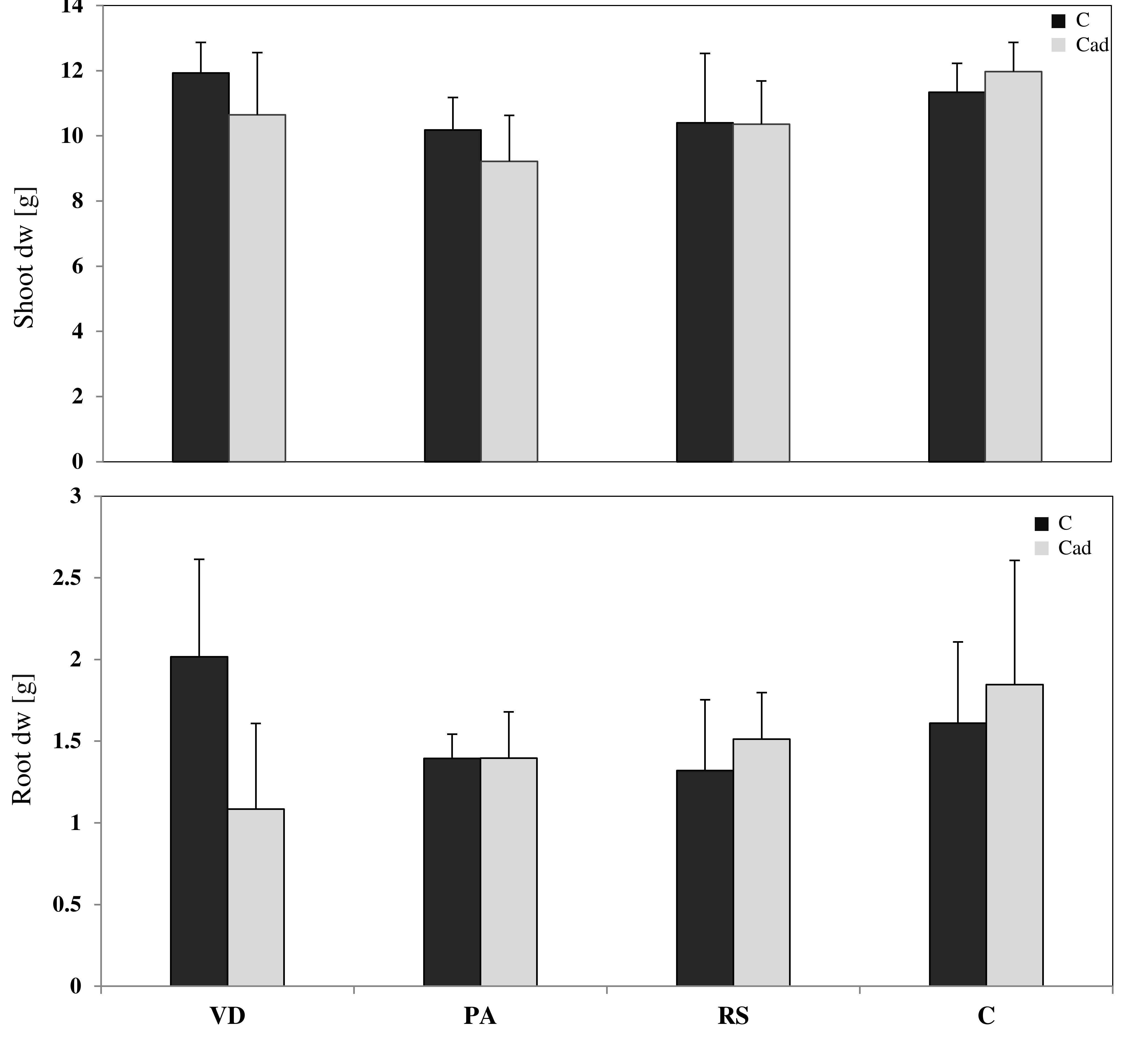

Supplement: fiz164_Supplement_Files [file fiz164_supplement_files.zip › Supplementary 4s.tif]

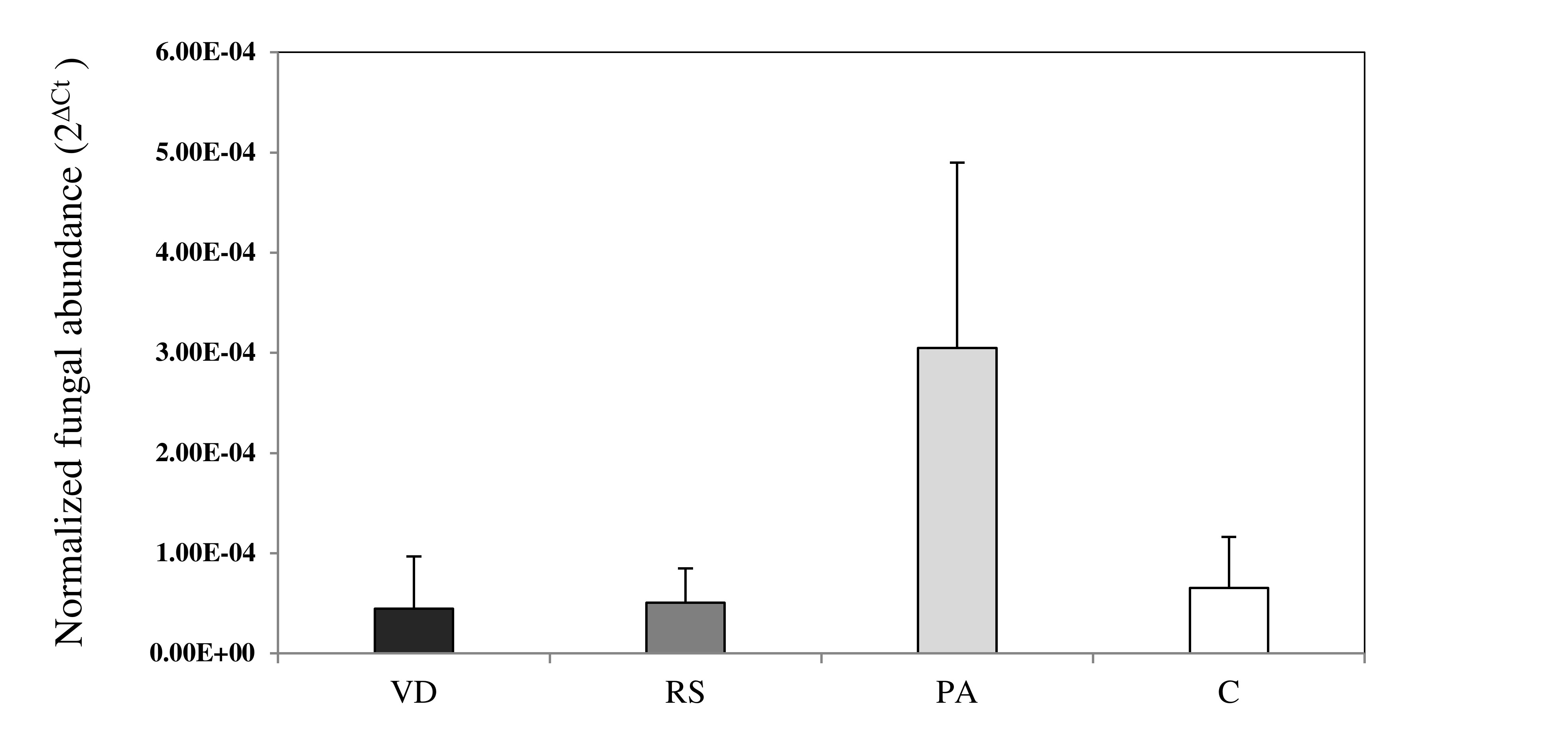

Supplement: fiz164_Supplement_Files [file fiz164_supplement_files.zip › Supplementary 6s.tif]
